# Computational modelling of movement-related beta-oscillatory dynamics in human motor cortex^☆^

**DOI:** 10.1016/j.neuroimage.2016.02.078

**Published:** 2016-06

**Authors:** Mrudul B. Bhatt, Stephanie Bowen, Holly E. Rossiter, Joshua Dupont-Hadwen, Rosalyn J. Moran, Karl J. Friston, Nick S. Ward

**Affiliations:** a Sobell Department of Motor Neuroscience and Movement Disorders, UCL Institute of Neurology, Queen Square, London WC1N 3BG, UK; b Virginia Tech Carilion Research Institute and Bradley Department of Electrical & Computer Engineering, Roanoke, VA, USA; c The Wellcome Trust Centre for Neuroimaging, Institute of Neurology, University College London, London, UK

**Keywords:** Beta, Oscillations, Movement-related beta desynchronisation, DCM, MEG, Primary motor cortex, M1

## Abstract

Oscillatory activity in the beta range, in human primary motor cortex (M1), shows interesting dynamics that are tied to behaviour and change systematically in disease. To investigate the pathophysiology underlying these changes, we must first understand how changes in beta activity are caused in healthy subjects. We therefore adapted a canonical (repeatable) microcircuit model used in dynamic causal modelling (DCM) previously used to model induced responses in visual cortex. We adapted this model to accommodate cytoarchitectural differences between visual and motor cortex. Using biologically plausible connections, we used Bayesian model selection to identify the best model of measured MEG data from 11 young healthy participants, performing a simple handgrip task. We found that the canonical M1 model had substantially more model evidence than the generic canonical microcircuit model when explaining measured MEG data. The canonical M1 model reproduced measured dynamics in humans at rest, in a manner consistent with equivalent studies performed in mice. Furthermore, the changes in excitability (self-inhibition) necessary to explain beta suppression during handgrip were consistent with the attenuation of sensory precision implied by predictive coding. These results establish the face validity of a model that can be used to explore the laminar interactions that underlie beta-oscillatory dynamics in humans *in vivo*. Our canonical M1 model may be useful for characterising the synaptic mechanisms that mediate pathophysiological beta dynamics associated with movement disorders, such as stroke or Parkinson's disease.

## Highlights

• This work aims to bridge the gap in understanding between neuronal oscillations measured with MEG and laminar interactions within motor cortex.
• We adapted a cortical microcircuit model used in sensory DCM studies to build a plausible model of laminar interactions in primary motor cortex.
• The proposed M1 model showed significantly more model evidence in explaining MEG data from M1 in humans than the inherited model.
• We replicated the in vitro finding of a dominant pathway from superficial to deep cortical layers at rest, providing face validity for our method.
• We show novel characterisation of laminar interactions underpinning beta-oscillatory dynamics within M1 during volitional movement in humans.

## Introduction

There is increasing interest in studying oscillations as a marker of brain function. Neuronal oscillations in the beta frequency range (15–30 Hz) in primary motor cortex (M1) are fundamental for motor control (Engel and Fries, 2010) and are a putative biomarker of pathophysiology in conditions like Parkinson's disease.

Magnetoencephalography (MEG) studies have shown that voluntary movement is associated with a systematic reduction in power of beta oscillations (movement-related betadesynchronisation, MRBD) in M1, which rebounds following movement cessation (post-movement beta rebound, PMBR) (Salmelin and Hari, 1994). The characteristics of beta oscillations change with healthy ageing (Rossiter et al., 2014a) and in disease states such as stroke (Rossiter et al., 2014b) and Parkinson's disease (Brown, 2006). An understanding of the mechanisms underlying these changes may therefore provide novel therapeutic opportunities (Ward, 2015a, Ward, 2015b).

In this paper, we show how a biophysical (neuronal mass) model facilitates the investigation of the laminar interactions underlying noninvasive measurements of neuronal oscillations from primary motor cortex (M1) in humans. Insights into cortical microcircuit dynamics in M1 to date have come from in vitro intra- and extracellular recordings in animals. From this work, the dominant (interlaminar) pathway in the cortical column appears to be from superficial to deep pyramidal cell layers (Weiler et al., 2008). Excitation of the deep pyramidal layer (Yamawaki et al., 2008) or possibly synchronous hyperpolarisation of superficial and deep pyramidal layers (Lacey et al., 2014) gives rise to beta oscillations. In both cases, recurrent interactions with inhibitory interneurons are important, as is the case for gamma oscillations (Traub et al., 2001).

We applied dynamic causal modelling (DCM) to MEG data acquired from humans. We focused on the spectral characteristics of a single source within M1 in order to model underlying neuronal activity in terms of specific cell populations within a typical motor cortical column. DCM then allows one to infer (i.e., estimate) synaptic and connectivity parameters associated with neuronal these sources (Moran et al., 2008, Moran et al., 2011, Moran et al., 2013).

Recently, advances have been made in DCM towards developing a canonical (repeatable) microcircuit model. This model has been developed under the dual constraints of complying with known intrinsic architecture within microcircuits and, at a functional level, is consistent with the computational anatomy of hierarchical Bayesian filtering (e.g., predictive coding) (Bastos et al., 2012). The nature of this model means it can explain cortical dynamics over a wide range of sensory brain areas (Bastos et al., 2012). However, the canonical microcircuit (CMC) model may not be appropriate for M1, given the cytoarchitectonic differences between such areas and M1 (Shipp, 2005). Our primary goal was to construct a canonical model taking account of known M1-specific microstructural characteristics and then, through Bayesian model comparison, determine the specific model architecture most likely to account for movement-related alterations in measured beta-band oscillations from M1. We used the ensuing model to examine laminar connectivity in human M1 (in comparison to known findings from rodent M1) and, in particular, test for a dominant descending excitatory drive through the connection from superficial to deep pyramidal layers at rest, empirically observed in previous animal studies (Weiler et al., 2008). Finally, we explored alterations in laminar connectivity during movement-related changes in beta oscillations. This series of analyses establish the face and construct validity of a canonical model for M1 activity that we hope will be useful in future DCM studies of pathophysiology, particularly in conditions that are associated with abnormal beta dynamics.

## Methods

### Participants

Eleven healthy participants took part (mean age 24.7 ± 1.6 years, 7 female, 2 left handed). Full written consent was obtained from all participants in accordance with the Declaration of Helsinki. The study was approved by the Joint Ethics Committee of the Institute of Neurology, UCL and National Hospital for Neurology and Neurosurgery, UCL Hospitals NHS Foundation Trust, London.

### Motor task

Participants performed visually cued dominant hand isometric handgrips using a force sensitive manipulandum during simultaneous MEG and electromyography (EMG) recording. Maximal voluntary contraction was recorded prior to scanning and subjects were then asked to perform visually cued handgrips at 30% MVC. Subjects performed 60 handgrips lasting 4 s each with an interstimulus interval ranging between 4 and 7 s.

### MEG recording

MEG signals were recorded during the handgrip task from a whole-head CTF Omega 275 MEG system (CTF, Vancouver, Canada), at a sampling rate of 600 Hz. Pre-processing of the data were performed offline in SPM12 (Wellcome Trust Centre for Neuroimaging, www.fil.ion.ucl.ac.uk/spm) (Litvak et al., 2011). Data were filtered from 2 to 100 Hz and epoched from − 1 s to + 5 s, where time 0 indicated the onset of visual cue. Artefacts from eye blinks and muscle contractions were identified by visual inspection, and corrupted trials were excluded from analysis. Power-line artefacts at 50 Hz were estimated and subtracted from the data, and epochs containing artefacts were removed with a semi-automatic artefact rejection protocol, based on a variance threshold.

### Data processing and analysis

To extract the spectral activity of M1 for subsequent dynamic causal modelling, we first estimated source activity in M1 using standard beamforming procedures: lead fields were computed using a single shell model, with a template inner skull canonical mesh being affine-transformed to fit MEG fiducials (nasion, left, and right pre-auricular).

Beta-band (15–30 Hz) power changes were localised using the linearly constrained maximal variance (LCMV) beamformer (Hillebrand and Barnes, 2005). This method projects sensor data using a linearly spatial filter derived from the lead-field of the source of interest and data covariance. The data covariance matrix was computed using three conditions (Rest, Mid-Grip and Post-Grip). The Rest time window was taken from − 1 s to 0 s with 0 as the onset of the visual cue to move. The Mid-Grip time window was from 1.0 s to 2.0 s following the visual cue onset. The Post-Grip time window was from 4 s to 5 s following the visual cue onset. In short, we used the same (global) filter (Brookes et al., 2008) to estimate the induced responses in three distinct time windows.

Volumetric statistical parametric maps (SPMs) of the t-statistic were computed for each subject using a grid spacing of 10 mm. At each location, the source orientation was taken to be in the direction yielding maximal signal variance. The source signal was then extracted from the location of peak change in beta power (15–30 Hz) within the primary motor cortex contralateral to the moving (dominant) hand (Sekihara et al., 2004). From these t-statistic images, we extracted the source signal from the location of peak change in beta power (15–30 Hz) within the primary motor cortices contralateral to the moving hand. Morlet-wavelet time–frequency analysis was used to explore the changes in beta across a trial from these locations, data were epoched again in order to visualise changes before and after the movement using the time window − 1 s to + 5 s. The spectrograms were rescaled in order to show percentage change from baseline (− 1 to 0 s) and averaged across trials.

The extracted data were then treated as a ‘virtual electrode’, from which data could be modelled in 5–45 Hz frequency range. The data were re-epoched from − 1 s to 0 s (rest), 1 s to 2 s (grip), 4 s to 5 s (post-grip), and concatenated (Barnes et al., 2004). Data were truncated between 5 and 45 Hz after inspection showed the majority of behaviourally tied spectral changes occurred within this range.

### Dynamic causal modelling (DCM)

**Fig. 1:**
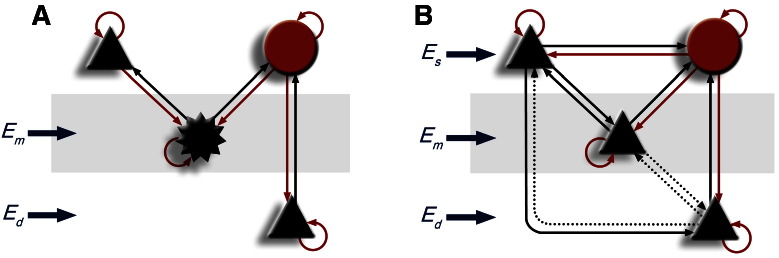
(A) Reduced CMC model before adaptations for M1. Pyramidal populations are shown as black triangles, the inhibitory subpopulation as a red circle, with the star representing spiny stellate cells. (B) M1 model space. Solid lines represent connections highly likely to be present based on the anatomical literature. Pyramidal populations are shown as black triangles and the inhibitory subpopulation as a red circle. Black lines represent glutamatergic projections, red lines represent GABAergic projections. Dotted lines represent connections investigated using model comparison. All combinations of dotted connections were tested with Bayesian model comparison. Es: Superficial input, Em: Middle input, Ed: Deep input; all inputs are scaled mixtures of pink and white noise to form biophysically plausible inputs.

Biophysical DCMs of canonical cortical microcircuits are used to infer synaptic mechanisms that underlie event or induced responses—or changes in the spectral characteristics of neural oscillations (Moran et al., 2009, Moran et al., 2011). Dynamic causal models of this sort allow empirical data from invasive (e.g., LFP/ECoG) or noninvasive (M/EEG, fMRI, NIRS) recordings to be used to characterise the neuronal interactions and architectures that generated them. This approach has been validated using local field potentials recorded in animal preparations where independent pharmacological/microdialysis assays have served to corroborate the modelling results (Moran et al., 2011). To date, the canonical microcircuit used in this type of DCM has been based largely on the known laminar architecture and intrinsic connectivity of sensory cortex and has been used in studies of primary visual cortex (V1) (Fig. 1A), (Moran et al., 2009, Bastos et al., 2012).

Here, we wish to study synaptic mechanisms in primary motor cortex (M1). The laminar architecture of M1 differs substantially from that of V1. M1 is mostly described as agranular, containing cell types with differing electrophysiological characteristics (Brodmann and Garey, 1999); however, recent evidence has emerged of a functional layer 4 in mice at the layer 3/5 A border (Yamawaki et al., 2014). M1 also has different inputs (Shepherd, 2009) and different interlaminar connectivity to V1, with dominant superficial to deep interlaminar pathways (Anderson et al., 2010, Weiler et al., 2008, Yu et al., 2008). These connectivity architectures provide important constraints on the DCMs used to model observed responses. A full mathematical description of the use of microcircuit dynamics within a DCM framework can be found in Moran et al. (2011), and Friston et al. (2012). Here, we will review the basic ideas and challenges posed in the current setting.

In brief, the generative model used by DCM consists of two parts: (i) the neuronal model, which comprises differential equations that prescribe average ensemble dynamics, specifically mass firing rates and postsynaptic membrane potentials which are themselves dependent on defined connectivity and physiological parameters and describe activity at the mesoscopic level, and (ii) an observation model mapping these hidden neuronal states (source-level activity) onto observed electrophysiological responses at the sensor level. Here, given our extraction of a time series from a virtual electrode in M1 (using beamforming), this observation model is simply a gain parameter that controls the amplitude matching from model to real data.

The neuronal model is effectively a neural mass (state space) model based on differential equations that effectively model linear or nonlinear synaptic convolutions of presynaptic input within several coupled neuronal populations (i.e. neural masses). Here, each neuronal subpopulation was modelled with two transformations: the convolution of presynaptic input to generate postsynaptic depolarisation and the (nonlinear) transformation of depolarisation to spiking output. The first transformation (presynaptic spiking to postsynaptic potentials) is equivalent to convolving a synaptic alpha kernel (either inhibitory or excitatory) with incoming spikes. The latter (postsynaptic potentials to spiking) transformation is approximated using a sigmoid activation function, which models a nonlinear transformation of voltage to spike rate, averaged over an ensemble of neurons.

The precise architecture of this model is defined by the form of the differential equations and the parameters encoding connection strengths and synaptic time constants. Both the structure and values of these parameters are estimated (using Bayesian model selection and inversion respectively). Model identification and estimation involve computing a posterior distribution over models and their parameters, given empirical data. An important aspect of this modelling rests on the prior distributions over the model parameters, which constrain the ranges that they can take and the dynamics the model can generate or explain. The prior expectations (i.e. means) of the intrinsic connections (see supplementary appendix) were chosen to generate high-frequency activity in the superficial pyramidal cells, relative to the deep pyramidal cells. This was motivated by multiple neurophysiological studies that have observed this spectral dissociation between superficial and deep layers of the cortical column in different cortical areas (Bollimunta et al., 2011, Buffalo et al., 2011, Maier, 2010, Roopun et al., 2006, Smith et al., 2012, Van Kerkoerle et al., 2014 and Xing et al., 2012).

Oscillatory activity is modulated by activity at both excitatory and inhibitory synapses where the respective effects are modelled by intrinsic connectivity (gamma) parameters that mimic the effective strength of these synapses at an average level (Wilson and Cowan, 1972). In previous DCM studies, these quantities have been shown to reflect neurotransmitter density and synaptic efficacy (Moran et al., 2011, Moran et al., 2013). Parameters associated with connections originating from excitatory populations represent glutamatergic projections, and inhibitory populations target GABAergic receptors.

The model used in this analysis comprised four subpopulations, with three pyramidal layers spatially distributed in superficial, middle, and deep layers, with diffuse inhibitory connectivity throughout (Fig. 1B). This neural model shares the same mathematical framework as the model used by Moran et al. (2013) for DCM of visual cortex but had equations and parameters adapted to reflect differences between primary visual and motor cortex. This entailed changing connectivity priors, signal contributions from subpopulations, and differences in afferent inputs (Shepherd, 2009); as well as changes to free parameters to account for differences in motor cortex physiology (e.g., synaptic time constants).

While the motor cortex is described as agranular, cells at the layer 3/5 A border exhibit properties that suggest the presence of a functional layer 4 (Yamawaki et al., 2014). Motor cortex layer 3/5 A cells are at a comparable cortical depth to Layer 4 in sensory cortex, (Shepherd, 2009, Weiler et al., 2008, Yamawaki et al., 2014), and so we maintained the middle pyramidal cell population from the CMC model. Middle pyramidal cells in M1 also receive thalamic input similar to that of Layer 4 (Shepherd, 2009, Yamawaki and Shepherd, 2015) and project to superficial layers in a similar fashion to Layer 4 cells (Shepherd, 2009, Weiler et al., 2008, Yamawaki et al., 2014). The model contained distinct inputs to each layer, after recent findings showed that all three layers receive thalamic input as well as input from other cortical areas in mice (Hooks et al., 2013). Key model parameter priors are provided in the supplementary appendix. Decisions to include or remove connections between subpopulations were based on studies of intralaminar M1 architecture in rodents (Anderson et al., 2010, Brecht et al., 2004, Hooks et al., 2013, Weiler et al., 2008) and subsequent Bayesian model selection between biologically plausible variations, as described below.

Mathematically, the neural mass model comprises pairs of first-order linear differential equations for each subpopulation of form:

1. ${\dot{x}}_{v}=x_{I}$
2. ${\dot{x}}_{I}=\kappa U-2\kappa x_{I}-\kappa^{2}x_{v}$
3. $U=\gamma S\left(x_{vpresynaptic}\right)+E$

The column vectors Xv and Xi represent mean voltages and currents, respectively, with each element corresponding to a specific subpopulation. The equations model a convolution of a subpopulation's presynaptic input (U) to produce a postsynaptic response. The rate constant (k) is the inverse of a lumped parameter that accounts for all membrane and dendritic time delays. Interlaminar delays were modelled using a Taylor expansion of the system's Jacobian (Kiebel et al., 2006), where interlaminar delays had a prior expectations of 1 ms. Each subpopulation receives two presynaptic inputs, an endogenous component (E) which comprises a mixture of white and pink noise, and a scaled input of ensemble firing from laminar contributions from different subpopulations. These firing rates are transformed via a sigmoidal function (slope = 2/3) to model membrane depolarisation (Moran et al., 2009).

The intrinsic connectivity parameter priors (Gs) are extremely important parameters that control the time constants of neuronal transients (in ERP models) and have a profound effect on the spectral responses (in models of cross spectral density). This is because they play the role of rate or time constants. The particular values in the CMC model have been optimised over several iterations to produce the sorts of frequencies that are seen empirically. Extrinsic inputs into the cortical column are a combination of white and pink noise to represent a physiologically representative frequency profile of non-specific neuronal activity. Such frequency profiles are characterised by 1/f (pink) noise and are found ubiquitously within the brain (Bédard et al., 2006; Bédard and Destexhe, 2009, Allegrini et al., 2009).

The model outputs are a mixture of the depolarisation for each subpopulation. Due to the tangential orientation of pyramidal dendrites, as well as the large disparity in size and hence conductance characteristics of deep layer Betz cells in M1, the contribution of each subpopulation is optimised during model inversion or fitting. The implicit observer function also comprises channel noise and amplifier gain components. The prior contributions to measured signal were 20% for the superficial and middle pyramidal layers and 60% for the deep pyramidal layer, due to the morphological differences between Betz cells in deep layers and other pyramidal cells. These priors were selected for to represent differences in conductance between such cells. Contributions to measured signals are optimised during model inversion.

The model was then inverted given measured data from each participant, to reduce an estimate of (or posterior density over) and their model parameters. This inversion uses a standard variational Laplace scheme to minimise free energy (a bound to the log model evidence) as described in (Friston and Stephan, 2007). The ensuing variational free energy provides approximation to log model evidence that is used for comparing different models.

### Bayesian model comparison

Model comparison enables one to evaluate the evidence for one model relative to competing models. For a full description of Bayesian model comparison see (Friston and Penny, 2011). In brief, the procedure compares models using their free energy as a proxy for model evidence (Friston and Stephan, 2007). By comparing the degree to which different models minimise free energy, we may evaluate the best model in a given model space. Generally, a difference of three or more in the free energy (or log evidence) between two models is taken as strong evidence for one model over the other (i.e., a likelihood ratio of exp(3):1 or 20:1).

We used Bayesian model comparison to compare alternative biologically plausible model structures. An anatomically based model space was constructed, based on electrophysiological data from animal models (Anderson et al., 2010, Brecht et al., 2004, Hooks et al., 2013, Weiler et al., 2008). These core connections were present in all models and all possible combinations of other (biologically plausible) connections were considered (Fig. 1B). Whenever a connection was included in the model, we also allowed for condition-specific changes in the connection. We also tested our model against the CMC model described in Moran et al. (2013). Dynamic causal modelling was implemented using the SPM8 software in MATLAB, and the practical procedure is fully described in Litvak et al. (2011).

### Statistical analysis

Parameter estimates were generated by model inversion. Gamma parameters represent connection strength at rest, while beta parameters represent changes in connection strength between conditions. The beta parameters modelled in this case pertained to the change in relative connection strength from rest to grip and rest to post-grip.

Parameter estimates were evaluated across subjects, using a parametric approach. The significance of particular (changes in) connections was determined by one-sampled t-tests across subjects and correction for multiple comparisons was performed using the Benjamini and Hochberg false discovery rate (Benjamini and Wei, 1999). This assesses the strength of (changes in) connectivity in relation to intersubject variability.

## Results

### MEG source localisation and power changes

**Fig. 2:**
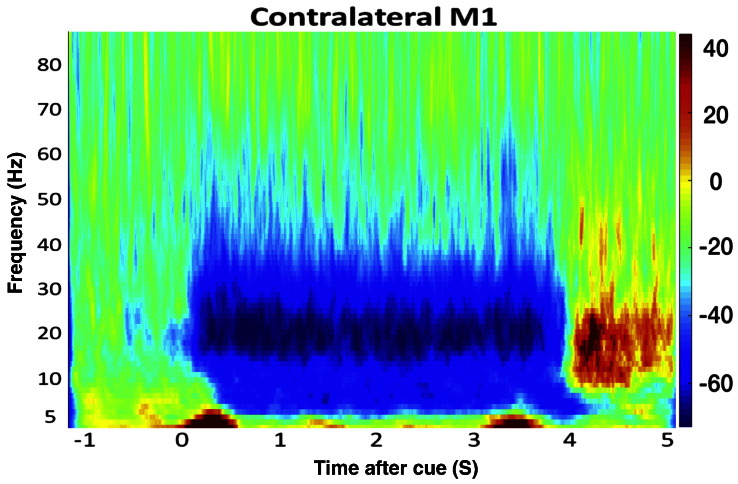
Group average time–frequency spectrogram showing changes in spectral power from primary motor cortex before, during and after hand grip for a single trial. The trial constituted a second of rest, followed by 4 s of grip, and 1 s post-grip and a jittered inter-trial interval of between 3 and 7 s. The visual cue to perform a hand grip was presented at 0 s. The colour indicates percentage change in power in comparison to rest (pre-grip) with red representing an increase, and blue a decrease in power compared to rest.

**Fig. 3:**
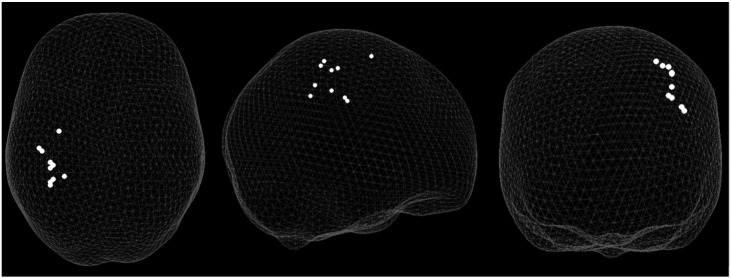
‘Glass brain’ showing the peak co-ordinates of beta (15–30 Hz) power change between rest and grip in contralateral sensorimotor cortex. Left-handed participants have had their MNI co-ordinates flipped in the sagittal plane to show all participants in the same hemisphere.

The time–frequency spectrogram averaged over all subjects is shown in Fig. 2. The location of the peak change in beta power for each subject is shown in Fig. 3. A clear desynchronisation of power is seen during grip, and restoration of beta power (above baseline) was seen following movement termination, as expected.

### Model selection

**Fig. 4:**
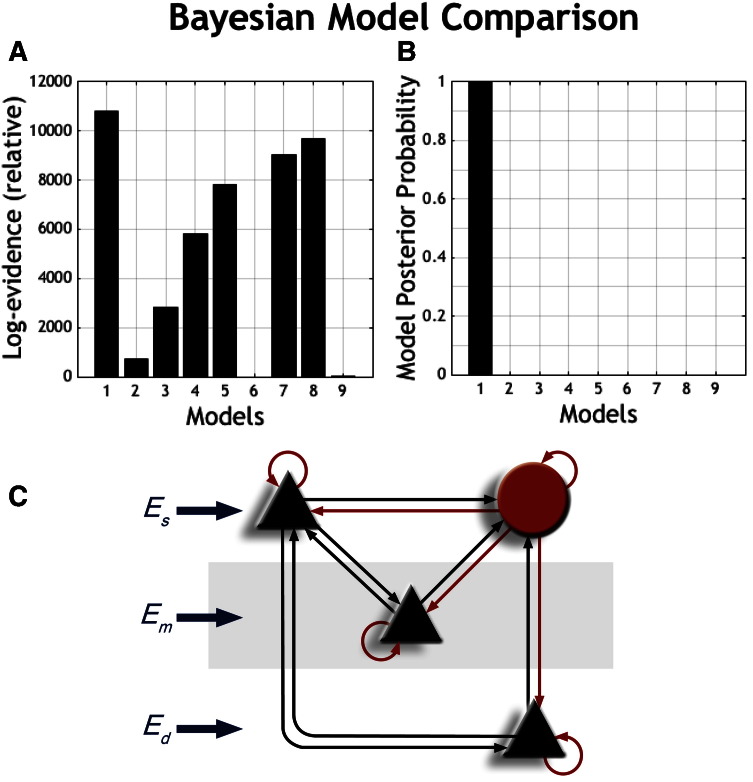
(A) Graphs showing a ranking of relative log evidences of the 8 M1 models (Models 1, 3–9 [Fig 1B]) and the reduced CMC model (Model 2 [Fig. 1A]) from which they were based. All tested M1 models had significantly more model evidence than the inherited reduced CMC model. (B) Posterior probability of winning M1 model. (C) Connectivity diagram of winning anatomical model based on log evidence (Model 1—Relative log evidence > 10,000) (Es: superficial input, Em: middle input, Ed: deep input; all inputs are scaled mixtures of pink and white noise to form biophysically plausible inputs.)

All configurations of connections were tested against each other, and the CMC model using Bayesian model comparison (Fig. 4A). We tested for a number of connections for which evidence in animal models was weak or absent. This analysis supported the inclusion of a connection from deep to superficial pyramidal layers, but not reciprocal connections between middle and deep layers. The metric used to appraise model space was log evidence. The interpretation of such a metric is dependent on the Bayes Factor (BF), whether providing weak (BF < 3), positive (3 ≤ BF < 20), strong (20 ≤ BF < 150), or very strong (BF ≥ 150) evidence for preferring one model over another is the basis for the calculation of model evidence. Strong evidence in favour of one model thus requires the difference in log evidence to be three or more (Penny et al., 2004). Bayes factor is simply the exponential of the difference in log evidences. The winning model, with 14 connections, had a relative log evidence of over 10,000 (Fig. 4B, 4C) (Kass and Raftery, 1995). The majority of motor cortex models had substantially more model evidence than the CMC model (Fig. 4A).

### Model parameter estimates

All further analyses were performed under the winning model shown in Fig. 4C. Intrinsic connections are those between populations of cells within the cortical column and their (gamma) parameters represent effective connection strength (i.e., log scaling relative to their prior values). The parameterisation of connectivity in the neural mass models used in DCM renders connections either excitatory or inhibitory. The parameters of the DCM determine the strength of (excitatory or inhibitory) connections by optimising these log scale parameters. In other words, the connection strength is modulated by the (positive) exponential of the parameter. This means a negative log scale parameter implies the connectivity is less than the prior mean (or weaker than expected), while a positive parameter means the connection strength is greater. In pyramidal populations, higher parameter estimates indicate stronger effective connections (the ability of one subpopulation to modulate the activity of another) that are excitatory in nature. Negative parameter estimates represent weaker excitatory pathways, not inhibitory pathways. Inhibitory pathways are modelled as separate projections from a distinct inhibitory subpopulation, where higher parameter estimates represent stronger inhibitory pathways, and negative inhibitory parameter estimates represent weaker inhibitory pathways.

**Fig. 5:**
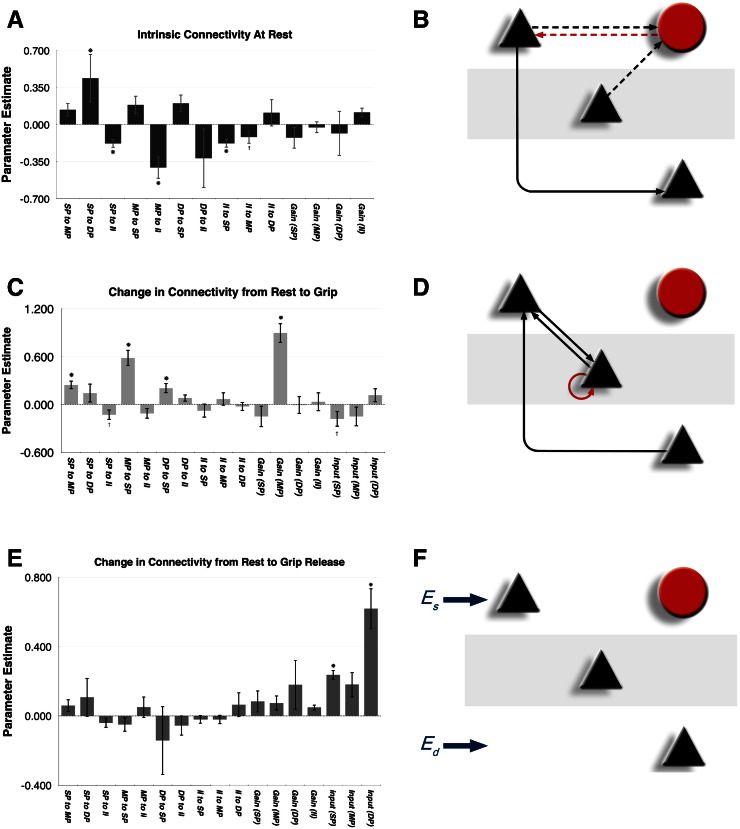
For A, C, E, asterisks represent statistically significant parameters across subjects as determined by a two-tailed t-test and multiple comparison correction using the Benjamini and Hochberg false detection rate (p < 0.05). Crosses represent parameters with statistical trends (0.05 < p < 0.10). (A) Parameter estimates for the intrinsic connectivity (gamma parameters) of winning model at rest. Gamma parameter estimates are (adimensional) log scaling coefficients with higher parameter estimates representing stronger effective connections. (B) Connectivity diagram showing statistically significant gamma parameter estimates (overall connection strength at rest). Solid lines represent positive parameter estimates while dotted lines represent negative parameter estimates. The dominant pathway from superficial to deep cells replicates dynamics previously measured in animal studies. (C) Parameter estimates for modulation of connection strength (beta parameters) during grip in comparison to rest (Beta1). Beta parameter estimates are adimensional log scaling with higher parameter estimates representing larger increases in connection strength with respect to rest. (D) Connectivity diagram showing statistically significant beta parameters (change in connection strength) from rest to grip. (E) Parameter estimates for modulation of connection strength (beta parameters) post-grip in comparison to rest (Beta2). (F) Connectivity diagram showing statistically significant beta parameters (change in connection strength) from rest to grip. Solid lines represent parameter estimate increases while dotted lines represent parameter estimate reductions. SP: Superficial layer, MP: middle layer, DP: deep layer, II: inhibitory interneurons.

The results for intrinsic connectivity are shown in Fig. 5A and B. A significant positive parameter estimate (strong pathway) was seen for the superficial to deep pyramidal layers. Negative parameter estimates (weaker pathways) were seen for the reciprocal connections between the inhibitory and superficial pyramidal subpopulations and for the middle pyramidal to inhibitory subpopulation connection. Full parameter means and statistics are included in the supplementary appendix.

We then went on to examine how connection strength was altered during and after handgrip. Our results demonstrated that in comparison to rest, hand grip resulted in an increase in the connection from deep to superficial pyramidal layers, as well as the reciprocal connections between superficial and middle pyramidal layers, together with an increase in the middle layer self connection (Fig. 5C, D). In comparison to the resting state, release of handgrip resulted in an increase of input into superficial and in particular, deep layers (Fig. 5E, F).

## Discussion

In this study, we set out to create a biologically plausible model that can accurately reproduce measured oscillatory dynamics in primary motor cortex of human subjects. Furthermore, because the model features are neurobiologically motivated, this approach can provide a mechanistically meaningful explanation for oscillatory dynamics. Previously, the CMC model used for DCM of M/EEG (Bastos et al., 2012) has proven successful in modelling activity in visual cortex (Bastos et al., 2012, Bastos et al., 2015, Moran et al., 2013, Muthukumaraswamy et al., 2013). However, given the known differences between primary motor and visual cortex, we reasoned that a number of principled changes to the model architecture were required to allow data from M1 to be modelled appropriately.

The spiny stellate population in the CMC model was removed. A further pyramidal population was added in middle layers and the cortical depth function altered, as we required a middle pyramidal population to receive input. A middle population was retained, despite the widely held view that M1 is agranular and lacks a functional Layer 4. This position was motivated by recent findings describing a functional M1 layer 4 in mice (Yamawaki et al., 2014). The ensuing model comprised 3 pyramidal populations, in Layers 2/3, Layer 5 A, and Layer 5B and a population of inhibitory interneurons. Changes to the model parameterisation also reflected the different relative contributions of laminar-specific subpopulations to measured signal; i.e., from large Betz cells in deep layers of M1.

Evidence from Weiler et al., 2008 led us to retain the reciprocal connections between the superficial and middle layers. The inhibitory connections were altered (in relation to the CMC model) so that an inhibitory population was reciprocally connected to all three pyramidal populations. This enabled us to model distributed inhibitory interneurons in M1 (Tokuno, 2000). Distinct inputs into each lamina were modelled due to evidence in mice from Hooks et al., 2013, showing sensory thalamus targets layers 2/3, 5 A and motor thalamus targets L5B. In addition, these authors show inputs from other cortical areas (such as M2 and orbital cortex) also target these three layers.

The final change, motivated by literature, was the inclusion of a connection from the superficial subpopulation to the deep subpopulation. This was due to the well-documented dominant superficial to deep pathways present in M1 in animals (Anderson et al., 2010, Weiler et al., 2008, Yu et al., 2008).

Having established this basic motor cortex model, we investigated the presence of 3 hypothetical connections. These were reciprocal connections between the deep and middle pyramidal populations, and a connection from the deep to the superficial pyramidal populations. All combinations of these connections were investigated (see Fig. 1B). Bayesian model selection found evidence for the connection from deep to superficial layers, but not for connections between middle and deep layers. The model was also tested against the original microcircuit model upon which it was based (Fig. 1A), with the majority of models (including less competitive M1 models) having substantially more evidence than the original CMC model (Fig. 4A).

When examining the connectivity architecture of our winning model (Fig. 4C) at rest, we were able to show that the dominant connection in human M1 is from superficial to deep pyramidal layers. In mouse M1, the same connection from superficial to deep cortical layers accounts for nearly 1/3 of all synaptic current (Weiler et al., 2008, Shepherd, 2009). This striking similarity between human and mouse M1 provides some face validity for our noninvasive modelling approach. Further, given this connection was not present in the CMC model on which the M1 model was based, it is clear that its inclusion is essential for modelling motor responses. Our results in human M1 at rest also point to reduced excitatory drive from superficial and middle layers to inhibitory interneurons together with reduced inhibitory drive to the superficial pyramidal layer. A reduction in inhibitory drive to the middle population was observed with a statistical trend. It is difficult to directly compare this result with previous rodent work because inhibitory signalling has not been measured exclusively (Weiler et al., 2008); however, the pattern of connectivity seen in human M1 points to important excitatory dynamics at rest in middle and superficial layers.

An advantage of our approach is the ability to look at changes in connection strength during volitional behaviour. In human M/EEG studies, a change in motor state from rest to grip is associated with a decrease in M1 oscillatory power in the beta band. Our results suggest that this movement-related beta desynchronisation is mediated by a number of changes in laminar connectivity. We see a relative increase in deep to superficial pyramidal layer connectivity as well as an increase in excitation between middle and superficial pyramidal layers and a reduction in middle pyramidal layer gain. Other approaches utilising ECOG data have been able to track such dynamic connectivity changes with the use of data-driven modelling to investigate the mechanisms involved in seizure initiation and termination (Freestone et al., 2016). Techniques such as this and DCM allow one to noninvasively track connectivity during active behaviour, and allow fitting of the models to a participant's (or patient's) own neuroimaging data, potentially accounting for small individual differences that in turn leads to a better understanding of variability in functional outcomes, or therapeutic response. Such models can even be used to estimate responses to new network perturbations, such as those seen in deep brain stimulation.

In comparison to rest, the immediate period after grip is characterised by a rebound in M1 beta power (Salmelin and Hari, 1994). Our results suggest that during this post-movement beta rebound, there is a relative increase in external excitatory drive to superficial and deep pyramidal layers, likely to come from other areas connected to M1, such as premotor cortex or thalamus (although we are unable to distinguish between these using a single source model).

In attempting to explain these task-related changes in laminar interactions, one can appeal to the predictive coding framework, which proposes that the brain constantly updates an internal model of the world, using cues from external sensory stimuli in a Bayesian manner (Friston and Kiebel, 2009). In predictive coding, expectations about (i.e. best estimates of) states of the world are encoded by activity in deep pyramidal cells. These expectations are used to generate descending predictions of sensory inputs and ensuing prediction errors. Prediction errors arise through comparing predictions with sensory information which validate or invalidate expectations, where prediction errors are thought to originate from superficial pyramidal cells (Friston and Kiebel, 2009). In our task, expectations may correspond to target grip force, and prediction error may be thought of as the error associated with the somatosensory and proprioceptive feedback produced by the applied force. At the neural level, this involves feedforward connections from superficial layers and feedback connections from deep layers; updating expectations and revising prediction errors, respectively (Bastos et al., 2012, Bastos et al., 2015). In this context, our results are consistent with this recurrent message passing; with increased excitatory connectivity from middle and deep layers to superficial layers, and from superficial to deep layers. More specifically, during the grip task, one would expect prior expectations about the (consequences of) target force to dominate over prediction errors. In predictive coding, this corresponds to an attenuation of sensory precision, where precision corresponds to the weight-afforded prediction errors. This sensory attenuation is exactly consistent with the decrease in gain or sensitivity of middle pyramidal cells—as reflected by an increase in self-inhibition (and the increased backward influence of deep pyramidal cells on superficial pyramidal cells). Crucially, these changes in sensitivity and gain are sufficient to explain grip-related beta suppression; suggested that beta desynchronisation may be a useful proxy for sensory attenuation (or precision) associated with motor execution.

It is important to remember that we model neuronal ensembles and not single cells, and so the connection from deep to superficial layers could reflect deep-layer feedback to superficial layers of other cortical columns that, clearly, cannot be resolved from MEG data. Furthermore, we only modelled a single source, which places some limits on the mechanistic insights gained from this approach. Firstly, the model represents different neural subpopulations organised into ensembles, with model parameter estimates based on effective connectivity. As a result, one cannot determine the precise flow of information through the cortical column, but only infer directive coupling under a model that is informed by other experimental methods, or careful planning of the behavioural task. In addition, our results indicate that inputs from other motor network nodes influence the dynamics within cortical columns in M1 following movement cessation. To fully understand how other nodes influence oscillatory dynamics in motor cortex, a single area M1 model is insufficient. To understand distributed responses of the sort, we would have to extend our model to include microcircuits in other brain areas involved in the generation and termination of voluntary movement. Extensions of the sort have been recently been used to investigate spectral asymmetries in feedforward and feedback connections between areas V1, V2, V3, and V4, suggesting this approach is viable for motor networks (Bastos et al., 2015).

In summary, we have described a new canonical microcircuit model of the primary motor cortex (M1), which can be used for DCM. We have demonstrated that this model has substantially more evidence when explaining data acquired during motor performance than alternative model structures. We have used this model to noninvasively characterise the dynamics of human M1 during a simple motor task, in terms of underlying synaptic (effective) connectivity. Future work, using this approach, may allow us to investigate the biological processes underpinning pathological disease states, where beta oscillations have been shown to be altered in comparison to healthy populations during voluntary action (Rossiter et al., 2014a, Rossiter et al., 2014b, Brown, 2006). Modelling oscillations in these disease states may provide mechanistic insights and provide novel therapeutic targets, especially those that involve nuancing the postsynaptic gain of key pyramidal cell populations.

## Funding

This research was supported by the European Commission under the 7th Framework Program-HEALTH-Collaborative Project Plasticise (contract no. 223524) www.plasticise.eu (Dr. Rossiter), and The Wellcome Trust (Dr. Nick Ward), and doctoral training grants from the Medical Research Council (Mrudul Bhatt, Stephanie Bowen).

## Appendix A Supplementary data

Contained within this appendix are model parameter estimates and their associated statistics, individual participant estimates for significant connections, full model equations and a single subject model fit. The appendix also contains tables showing the model settings and prior values. Image 1
