# Supplementary material for: Computational modelling of movement-related beta-oscillatory dynamics in human motor cortex
Source: Neuroimage. 2016 Jun;133:224–32. doi: 10.1016/j.neuroimage.2016.02.078 (PMC4907685; doi:10.1016/j.neuroimage.2016.02.078)
Supplement: Supplementary file 1 — Contained within this appendix are model parameter estimates and their associated statistics, individual participant estimates for significant connections, full model equations and a single subject model fit. The appendix also contains tables showing the model settings and prior values. [file mmc1.pdf]

SUPPLEMENTARY APPENDIX

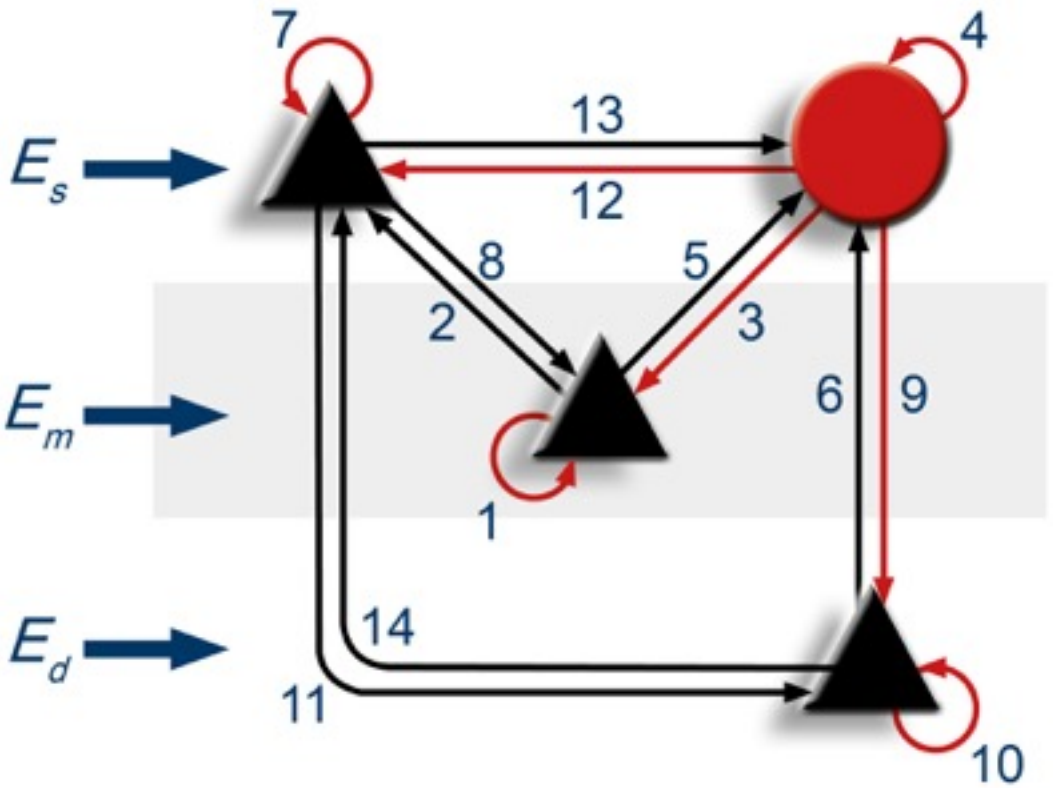

Superficial Pyramidal

$$\dot{x}_{v_{sp}} = x_{I_{sp}}$$

$$\dot{x}_{I_{sp}} = \kappa[(\gamma_8 + \beta_8)S(x_{v_{mp}}) + (\gamma_{14} + \beta_{14})S(x_{v_{dp}}) - (\gamma_7 + \beta_7)S(x_{v_{sp}}) - (\gamma_{13} + \beta_{13})S(x_{v_{ii}})] - 2\kappa x_{I_{sp}} - \kappa_v^2 x_{v_{sp}} + u$$

Middle Pyramidal

$$\dot{x}_{v_{mp}} = x_{I_{mp}}$$

$$\dot{x}_{I_{mp}} = \kappa[(\gamma_2 + \beta_2)S(x_{v_{sp}}) - (\gamma_3 + \beta_3)S(x_{v_{ii}}) - (\gamma_1 + \beta_1)S(x_{v_{mp}})] - 2\kappa x_{I_{mp}} - \kappa_v^2 x_{v_{mp}} + u$$

Deep Pyramidal

$$\dot{x}_{v_{dp}} = x_{I_{dp}}$$

$$\dot{x}_{I_{dp}} = \kappa[(\gamma_{11} + \beta_{11})S(x_{v_{sp}}) - (\gamma_{10} + \beta_{10})S(x_{v_{dp}}) - (\gamma_9 + \beta_9)S(x_{v_{ii}})] - 2\kappa x_{I_{dp}} - \kappa_v^2 x_{v_{dp}} + u$$

Inhibitory Interneurons

$$\dot{x}_{v_{ii}} = x_{I_{ii}}$$

$$\dot{x}_{I_{ii}} = \kappa[(\gamma_5 + \beta_5)S(x_{v_{mp}}) + (\gamma_6 + \beta_6)S(x_{v_{dp}}) + (\gamma_{12} + \beta_{12})S(x_{v_{sp}}) - (\gamma_4 + \beta_4)S(x_{v_{ii}})] - 2\kappa x_{I_{ii}} - \kappa_v^2 x_{v_{ii}} + u$$

Figure 1 (Appendix) - Graphical representation of all connections in winning model. Each connection is numbered in accordance with its connectivity parameter number. Paired differential equations are shown for each subpopulation. These equations account for all connections entering into a subpopulation and the biophysical natures of such connections. Each connection has a gamma (intrinsic connectivity) and beta (modulation of intrinsic connectivity due to experimental intervention) are convolved with sigmoid functions that represent relevant physiological parameters associated with the subpopulations from where the connections originate. The equations represent membrane delay constants and other physiological parameters using the inverse of a lumped parameter (k). Finally each subpopulation is driven by an Input (u).

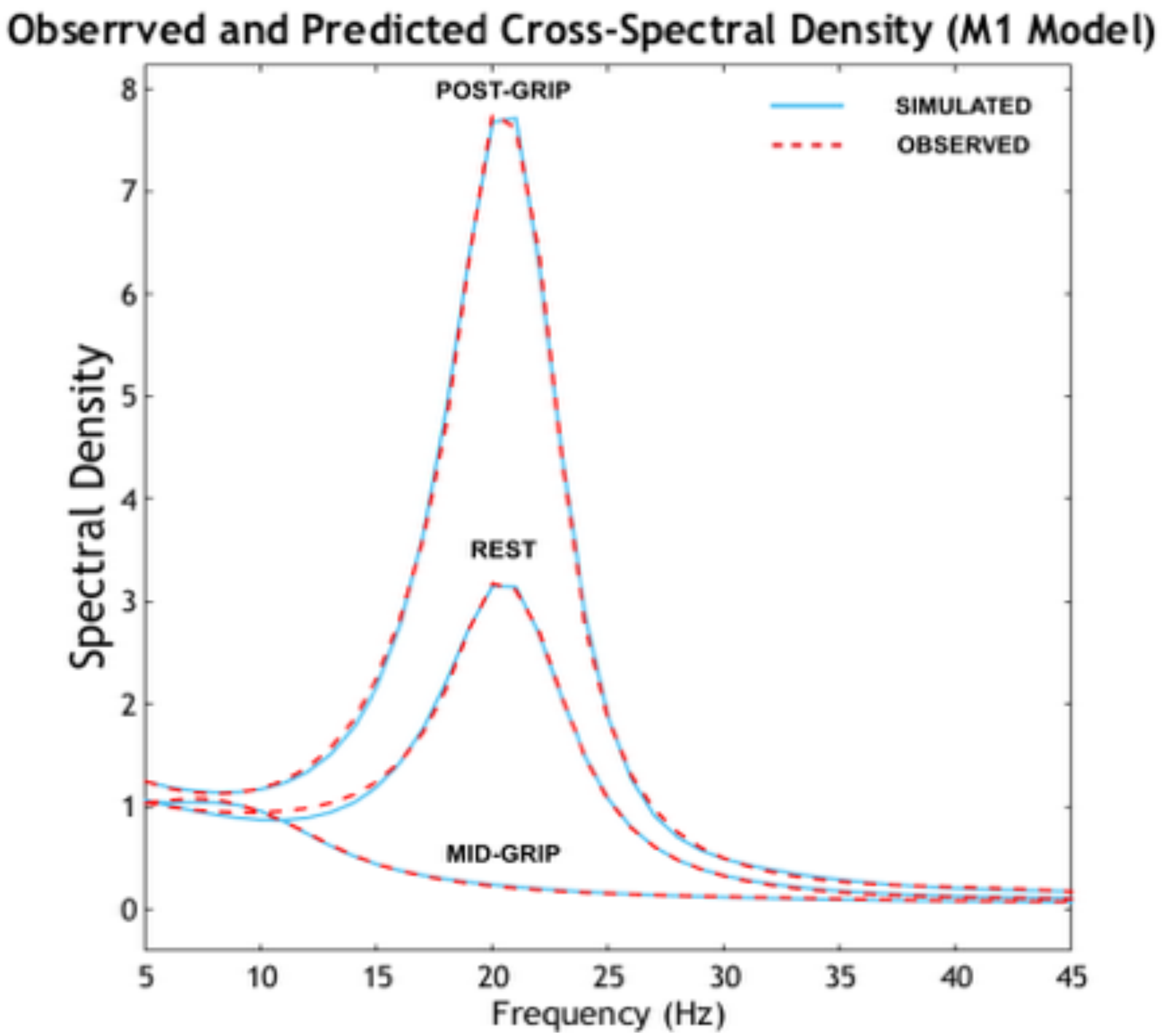

Figure 2 (Appendix) - Measured and simulated spectral profiles for a single subject during rest, mid-grip and post-grip. The lines show the spectral profiles before, during and after handgrip. Dotted lines represent measured data and solid lines represent model predictions.

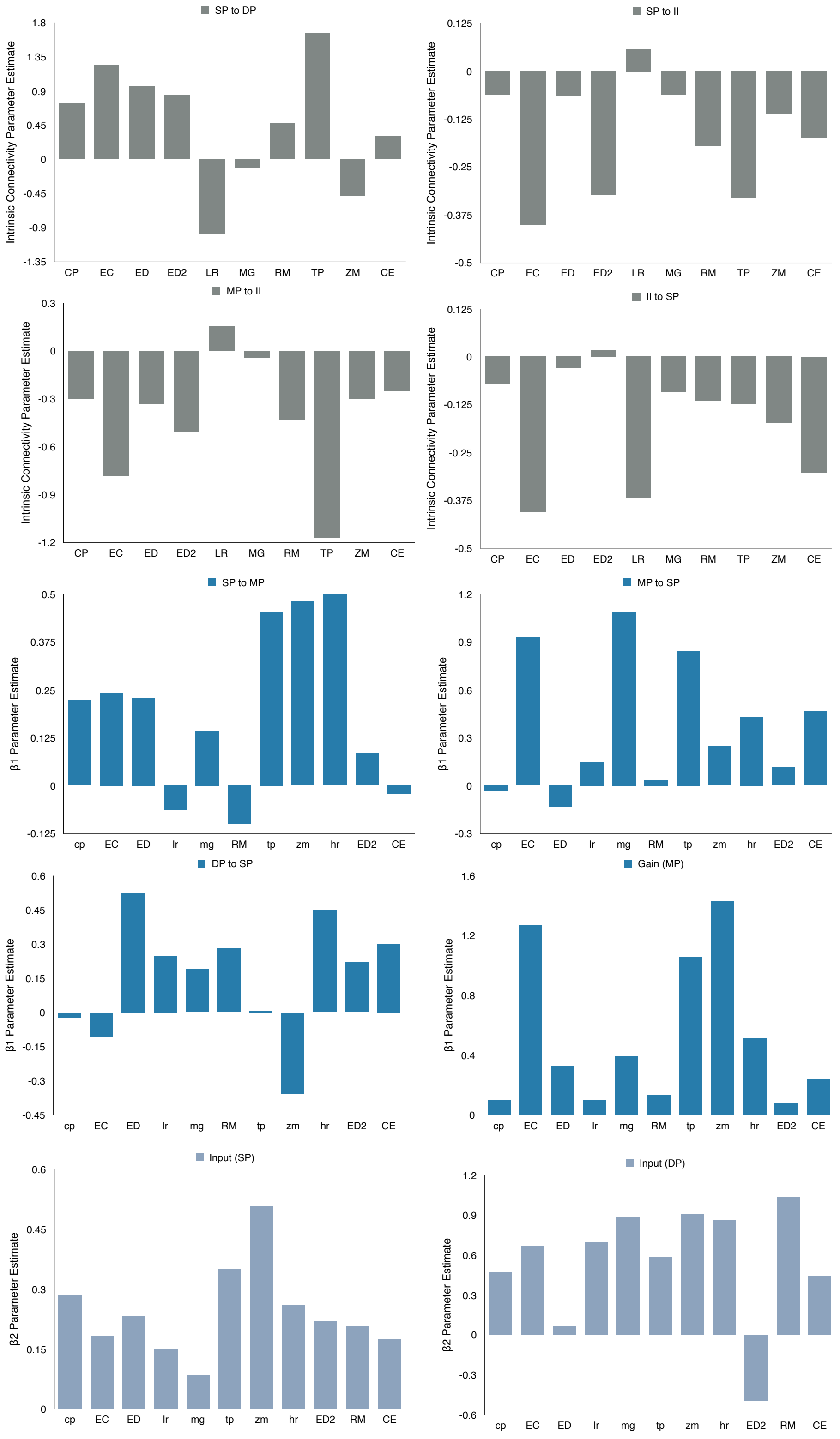

Figure 3 (Appendix) - Individual subjects and parameter estimates for statistically significant connectivity parameters. In grey are intrinsic connectivity parameters, in blue are β1 parameters, and in lilac are β2 parameters.

**Connectivity Parameter number**	**Parameter representation**	**Abbreviation**
**1**	Middle Pyramidal gain	**Gain (mp)**
**2**	Superficial pyramidal connection to Middle pyramidal population	**sp2mp**
**3**	Inhibitory connection to superficial pyramidal population	**ii2sp**
**4**	Inhibitory population gain	**Gain (ii)**
**5**	Middle pyramidal connection to Inhibitory population	**mp2ii**
**6**	Deep pyramidal connection to Inhibitory population	**dp2ii**
**7**	Superficial Pyramidal gain	**Gain (sp)**
**8**	Middle pyramidal connection to Superficial pyramidal population	**mp2sp**
**9**	Inhibitory connection to Deep pyramidal population	**ii2dp**
**10**	Deep Pyramidal gain	**Gain (dp)**
**11**	Superficial pyramidal connection to Deep pyramidal population	**sp2dp**
**12**	Superficial pyramidal connection to inhibitory population	**sp2ii**
**13**	Inhibitory connection to Superficial pyramidal population	**ii2sp**
**14**	Deep pyramidal connection to superficial pyramidal population	**dp2sp**

Table 1 (Appendix) - connectivity parameters, their biological interpretation and abbreviations

Parameter	*Prior Mean (mu)*	*Prior Variance (σ)*	*Physiological Interpretation*
**OBSERVATIONAL MODEL**			
Alpha(u)	***0***	***1/16***	Exogenous white input
Alpha(s)	***0***	***1/16***	Channel white noise
Beta(u)	***0***	***1/16***	Exogenous pink input
Beta(s)	***0***	***1/16***	Channel pink noise
Theta	***1***	***1***	Lead-field gain
[Superficial, Middle, Deep]	***[0.2, 0.2, 0.6]***	***[1/16]***	Subpopulation signal contribution
**NEURONAL SOURCES**			
1/k1	***8 ms***	***1/16***	Time constant (Superficial pyramidal)
1/k2	***8 ms***	***1/16***	Time constant (Middle pyramidal)
1/k3	8 ***ms***	***1/16***	Time constant (Inhibitory interneurons)
1/k4	***8 ms***	***1/16***	Time constant (Deep pyramidal)
Gamma [1…14]	***[4, 4, 4, 4, 4, 2, 4, 4, 2, 1, 2, 4, 4, 2]***	***1/16***	Intrinsic connection parameters or 'connection strengths' priors.
Beta [1…14]	***0***	***1/8***	Modulation of respective gamma parameters. These pertain to changes in connection strength between conditions.
D	***1 ms***	***1/16***	Laminar delay.

Table 2 (Appendix) - Table showing free model parameters for the observation and neuronal model, their prior values and physiological interpretation.

INTRINSIC CONNECTIVITY	SP to MP	SP to DP	SP to II	MP to SP	MP to II	DP to SP	DP to II	II to SP	II to MP	II to DP	Gain (SP)	Gain (MP)	Gain (DP)	Gain (II)
MEAN	0.139	0.438	-0.179	0.185	-0.407	0.200	-0.318	-0.177	-0.119	0.110	-0.125	-0.026	-0.084	0.114
ADJUSTED P-VAL (BENJAMINI & HOCHBERG FDR)	0.244	0.046	0.042	0.244	0.046	0.244	0.496	0.042	0.254	0.596	0.496	0.758	0.758	0.244
SIGNIFICANT	FALSE	TRUE	TRUE	FALSE	TRUE	FALSE	FALSE	TRUE	FALSE	FALSE	FALSE	FALSE	FALSE	FALSE
TREND	FALSE	FALSE	FALSE	FALSE	FALSE	FALSE	FALSE	FALSE	FALSE	FALSE	FALSE	FALSE	FALSE	FALSE
STANDARD ERROR	0.063	0.227	0.040	0.086	0.105	0.083	0.281	0.040	0.062	0.128	0.103	0.055	0.212	0.044

Table 3 (Appendix) - Parameter means and associated statistics (at the between subject level) for Intrinsic Connectivity (gamma) parameters

Mid-Grip	SP to MP	SP to DF	SP to II	MP to SP	MP to II	DP to SP	DP to II	II to SP	II to MP	II to DP	Gain (SP	Gain (MP)	Gain (DP	Gain (II)	Input (SP	Input (MP	Input (DP
MEAN	0.245	0.144	-0.128	0.583	-0.112	0.205	0.082	-0.077	0.068	-0.024	-0.150	0.896	-0.006	0.034	-0.295	-0.351	0.055
ADJUSTED P-VAL (BENJAMINI & HOCHBERG FDR)	0.012	0.990	0.064	0.013	0.094	0.046	0.265	0.251	0.898	0.514	0.113	0.007	0.502	0.732	0.115	0.115	0.662
SIGNIFICANT	TRUE	FALSE	FALSE	TRUE	FALSE	TRUE	FALSE	FALSE	FALSE	FALSE	FALSE	TRUE	FALSE	FALSE	FALSE	FALSE	FALSE
TREND	FALSE	FALSE	TRUE	FALSE	TRUE	FALSE	FALSE	FALSE	FALSE	FALSE	FALSE	FALSE	FALSE	FALSE	FALSE	FALSE	FALSE
STANDARD ERROR	0.067	0.102	0.047	0.114	0.045	0.038	0.045	0.043	0.097	0.191	0.102	0.128	0.101	0.086	0.091	0.109	0.088

Table 4 (Appendix) - Parameter means and associated statistics (at the between subject level) for Mid-Grip (beta-1) parameters

Post-Grip	SP to MP	SP to DP	SP to II	MP to SP	MP to II	DP to SP	DP to II	II to SP	II to MP	II to DP	Gain (SP)	Gain (MP)	Gain (DP)	Gain (II)	Input (SP)	Input (MP)	Input (DP)
**MEAN**	0.059	0.107	-0.040	-0.050	0.050	-0.143	-0.056	-0.020	-0.020	0.064	0.084	0.074	0.179	0.048	0.242	0.156	0.558
**ADJUSTED P-VAL (BENJAMINI & HOCHBERG FDR)**	0.220	0.456	0.281	0.350	0.519	0.574	0.453	0.535	0.541	0.484	0.311	0.200	0.340	0.337	0.001	0.303	0.016
**SIGNIFICANT**	FALSE	FALSE	FALSE	FALSE	FALSE	FALSE	FALSE	FALSE	FALSE	FALSE	FALSE	FALSE	FALSE	FALSE	TRUE	FALSE	TRUE
**TREND**	FALSE	FALSE	FALSE	FALSE	FALSE	FALSE	FALSE	FALSE	FALSE	FALSE	FALSE	FALSE	FALSE	FALSE	FALSE	FALSE	FALSE
**STANDARD ERROR**	0.045	0.037	0.026	0.017	0.064	0.054	0.065	0.037	0.072	0.156	0.093	0.022	0.027	0.066	0.027	0.071	0.107

Table 5 (Appendix) - Parameter means and associated statistics (at the between subject level) for Post-Grip (beta-2) parameters
